# The Relationship Between the Striatal Dopaminergic Neuronal and Cognitive Function With Aging

**DOI:** 10.3389/fnagi.2020.00041

**Published:** 2020-02-28

**Authors:** Hongliang Li, Shigeki Hirano, Shogo Furukawa, Yoshikazu Nakano, Kazuho Kojima, Ai Ishikawa, Hong Tai, Takuro Horikoshi, Takashi Iimori, Takashi Uno, Hiroshi Matsuda, Satoshi Kuwabara

**Affiliations:** ^1^Department of Neurology, Graduate School of Medicine, Chiba University, Chiba, Japan; ^2^Department of Neurology, Japanese Red Cross Narita Hospital, Chiba, Japan; ^3^Department of Neurology, Chiba Rosai Hospital, Chiba, Japan; ^4^Department of Neurology, JR Tokyo General Hospital, Tokyo, Japan; ^5^Diagnostic Radiology and Radiation Oncology, Graduate School of Medicine, Chiba University, Chiba, Japan; ^6^Department of Radiology, Chiba University Hospital, Chiba, Japan; ^7^Integrative Brain Imaging Center, National Center of Neurology and Psychiatry, Tokyo, Japan

**Keywords:** dopamine transporter, SPECT, cognitive function, aging, Wechsler Adult Intelligence Scale, verbal function

## Abstract

Both cognitive function and striatal dopamine function decline by normal aging. However, the relationship among these three factors remains unclear. The aim of this study was to elucidate the association among age-related changes in the striatal dopamine transporter (DAT) and cognitive function in healthy subjects. The 30 healthy volunteers were enrolled in this research, the age ranged from 41 to 82 (64.5 ± 11.5, mean ± SD). All subjects were scanned with both T1-weighted magnetic resonance imaging (MRI) and ^123^I-FP-CIT single-photon emission computed tomography (SPECT) images. The Wechsler Adult Intelligence Scale-Third Edition (WAIS-III) was used to evaluate cognitive function. Six spherical regions of interest (ROI) using 10 mm in diameter on the caudate nucleus, anterior putamen and posterior putamen were manually drawn on MRI image which was applied onto SPECT image. The relationship between striatal occipital ratio (SOR) values and WAIS-III subscore were analyzed by multiple regression analysis. Subscores which was significant were further analyzed by path analyses. Full intelligence quotient (IQ), verbal IQ, verbal comprehension were all positively correlated with age-adjusted striatal DAT binding (*P* < 0.01). Multiple regression analyses revealed that the coding digit symbol correlated with all striatal regions except for the left caudate (*P* < 0.04). Picture completion and right caudate, similarities and left caudate also showed a positive correlation (*P* < 0.04). Path analysis found that the right caudate and picture completion; the left caudate and similarities were correlated independently from age, whereas the models of coding digit symbol were not significant. These results suggest that age-based individual diversity of striatal DAT binding was associated with verbal function, and the caudate nucleus plays an important role in this association.

## Introduction

In human cognitive performance peaks around age 20, and subsequently, as people age, a wide range of cognitive tasks exhibit a steady and accelerated decline (Salthouse, 2010). Cognitive functions that decline with normal aging include the speed of information processing (Verhaeghen and Salthouse, 1997), memory (Engle, 2002; Rieckmann et al., 2018), attention, and executive control (Miyake et al., 2000). Conversely, some cognitive functions, such as vocabulary and general information, remain stable throughout normal aging, at least until the seventh decade of life (Salthouse, 2010).

Dopamine plays an important role in complex cognitive functions such as working memory, cognitive flexibility, language and thought, motor planning, abstract representation, temporal analysis/sequencing, and generativity (Volkow et al., 1998; Previc, 1999; Mozley et al., 2001). The dopamine transporter (DAT) is a membrane-bound protein located on the presynaptic terminals that regulates intrasynaptic dopaminergic levels by re-uptaking dopamine; its radioligand serves as a biomarker for the presynaptic dopaminergic system (i.e., nigrostriatal and mesolimbic pathway). DAT imaging has been widely applied in both clinical and research settings. [^123^I]-2beta-carbometoxy-3beta-(4-iodophenyl)-N-(3-fluoropropyl) nortropane ([^123^I]FP-CIT; ioflupane) is a probe for single-photon emission computed tomography (SPECT), which enables us to quantify striatal DAT binding *in vivo* (Tatsch and Poepperl, 2013). Previous studies have shown that the presynaptic striatal dopamine function declines with normal aging (Kaasinen et al., 2015; Matsuda et al., 2018). Neurodegeneration in the nigrostriatal dopaminergic neurons occurs in Parkinson’s disease (PD), which presents as bradykinesia, rigidity, and resting tremor, accompanied by some nonmotor symptoms such as depression, autonomic dysfunction, and cognitive impairment. Dopamine has been postulated as one of the many pathological hypotheses for cognitive impairment in PD (Bosboom et al., 2004; Hirano et al., 2012; Biundo et al., 2016).

The Wechsler Adult Intelligence Scale (WAIS)-Third Edition consists of 14 subscore section tests including picture completion, vocabulary, coding digit symbol, similarities, block design, arithmetic, matrix reasoning, digit span, information, picture arrangement, comprehension, symbol search, letter-number sequencing, and object assembly (Kaufman and Lichtenberger, 2006). By calculating the subscores, one can estimate verbal comprehension (vocabulary, similarities, information, comprehension), working memory (arithmetic, digit span, letter-number sequencing), perceptual organization (picture completion, block design, matrix reasoning), processing speed (coding digit symbol, symbol search), and intelligence quotient (IQ).

The evidence of a correlational triad among normal aging, cognitive function, and striatal dopamine presynaptic function is still limited, and these relationships remain unclear. Mozley et al. scanned DAT imaging in 66 healthy subjects who were evaluated by customized cognitive tests and found that young women exhibited higher caudate DAT binding and higher language learning performance (Mozley et al., 2001). Twelve healthy subjects underwent DAT positron emission tomography and several cognitive batteries; the results showed that episodic memory and executive function were associated with striatal DAT binding and that both cognitive batteries declined with aging (Erixon-Lindroth et al., 2005). This report also demonstrated that the information subscore of the WAIS-revised was associated with inter-individual variance of striatal DAT binding. These findings should be extended by using standard cognitive tests and normalizing the data by controlling for age.

The aim of this study was to clarify the relationship between the striatal DAT function, evaluated by [^123^I]FP-CIT SPECT, and cognitive function measured by a standardized neuropsychological test, the WAIS-III, in healthy subjects. First, these relationships were investigated by age-normalized cognitive scores and age-adjusted striatal DAT binding. Next, the age-unadjusted striatal subregions were further explored with age-unadjusted cognitive values, followed by the evaluation of associations among age, DAT binding, and cognition, which were assessed using path analyses.

## Materials and Methods

### Participants

This study was approved by the ethical committee of Chiba University (#2074). All participants received explanations of the study orally and by documents and provided written informed consent. Thirty healthy Japanese subjects (21 females and 9 males) were enrolled in this study from April to December 2016; they were recruited using local advertisements. Exclusions for the subjects included: neurodevelopmental disorders; neurologic or psychiatric illnesses including Parkinsonism and rapid eye movement sleep behavior disorder and depression, magnetic resonance imaging (MRI) contraindications, and the use of medicine that influences the accumulation of [^123^I]FP-CIT in the brain (cocaine, mazindol, methylphenidate, and selective serotonin reuptake inhibitors). The participants received financial compensation after the study. The participants ranged in age from 41 to 82 years old [mean ± standard deviation (SD): 64.5 ± 11.5]. The educational duration ranged from 9 to 16 years (mean ± SD: 13.8 ± 2.1). Twenty-five subjects were right-handed, three subjects were left-handed, and two subjects were ambidextrous.

### Neuropsychological Assessment

All subjects underwent the mini-mental state examination (MMSE), frontal assessment battery (FAB), and WAIS-III. These neuropsychological tests were performed on the same day as the MRI scan. All tests were administered by an experienced clinical neuropsychologist and took from 90 to 120 min to complete. No participants reported a total MMSE score of less than 24. The WAIS-III includes 14 subtests, of which 13 were administered (the object assembly subtest was omitted since it does not contribute to the full-scale IQ; Cockcroft et al., 2015). Thirteen age-adjusted subscores were further calculated for verbal comprehension, working memory, perceptual organization and processing speed, Performance IQ (PIQ), Verbal IQ (VIQ), and the full score IQ (FIQ), which is calculated from PIQ and VIQ. The 13 age-unadjusted raw subscores and age-adjusted subscores were both used in these analyses.

### Image Acquisition

#### MRI

All MR scans were performed using a 3.0T Discovery MR750 (GE Healthcare, Milwaukee, WI, USA). T1-weighted images were collected for the purpose of placing the region-of-interest (ROI). T1 images were obtained by sagittal 3-D fast spoiled gradient-echo (FSPGR) sequences. The following parameters were used for the T1-weighted images: repetition time, 6.4 ms; echo time, 2.6 ms; inversion time, 420 ms; flip angle, 15°; field-of-view, 240 × 240 mm; matrix, 256 × 256; slices, 178; slice thickness, 1.4 mm; overlap, 0.7 mm; and scan time, 214 s. Transaxial fluid attenuation inversion (FLAIR) imaging was also conducted for screening purposes. No intracranial abnormal structures were identified in any of the participants.

#### [^123^I]FP-CIT SPECT

A SPECT scan was conducted within three months after the MRI screening. A dose of 167 MBq [^123^I]FP-CIT was injected intravenously while the subjects were at rest. Image acquisition was performed 3.5 h after the injection, with Infinia + Hawkeye4 (GE Healthcare, Milwaukee, WI, USA) mounted with extended low energy general-purpose (ELEGP) collimators. Projection data were acquired for 30 min. The data were reconstructed by the ordered subset expectation maximization (OSEM) method (iteration: 5, subset: 10). Images were acquired in a 128 × 128 matrix of 2.95-mm thick axial slices under continuous rotation (8 rotations, 3 min per rotation) in 4-degree steps and were reconstructed with a Butterworth filter (cut-off frequency 0.5 cycle/cm, order 8). Attenuation was corrected using Chang’s technique (coefficient: 0.07).

### Image Analysis

#### Automated Striatal DAT Binding Adjusted by Age

The specific binding ratios (SBRs) of the DAT-SPECT values were calculated using DaT View version 6.1 software (AZE Ltd., Kanagawa, Japan) based on Bolt’s method (Tossici-Bolt et al., 2006; Miyai et al., 2015). In brief, the ROI was automatically placed on the striatum. A reference ROI was automatically selected outside the striatal ROI in the cerebral cortex. Fully automated detection relies on a 50% iso-contour based on the non-striatum maximum uptake. This automated procedure gives a consistent ROI size in both normal and abnormal scans (Tossici-Bolt et al., 2006). The right and left striatal SBR was defined as the ratio of the concentrations of specific (striatum) to non-specific (cerebral cortex) radioligand binding. SBR was corrected by phantom experiment, following adjustment for age using 256 healthy subjects, and was called the age-adjusted specific binding ratio (SBR_adj_; Tossici-Bolt et al., 2006; Matsuda et al., 2018). The advantages of using the SBR_adj_ method are that the placement of the ROI is automatic without investigator bias and age adjustment is performed using a large normal database. The drawback of this software is that subregions of the striatal ROI cannot be investigated.

#### Manually Placed ROI in Striatal Subdivision

To investigate DAT activities in the striatal subregions, manual ROI analyses were conducted. AquariusNET version 4.4 (TeraRecon, CA, USA) was utilized to display, fuse images, and place ROIs on 3D-T1-weighted MRI and [^123^I]FP-CIT SPECT images. The standard MRI position was adjusted to the anterior commissure-posterior commissure line. The SPECT images were automatically coregistered with MRI in the same space. Two continuous transverse slices including the striatum were selected for further analyses.

**Figure 1:**
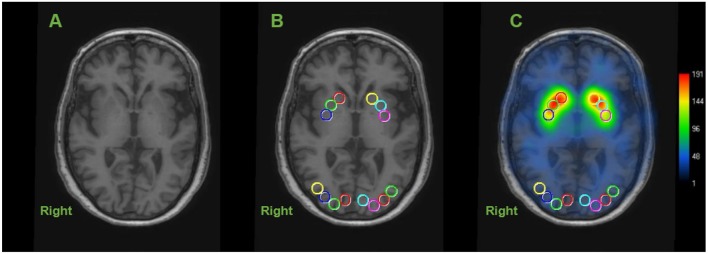
Representative image of regions of interest (ROIs) in dopamine transporter (DAT) image. Circular ROIs were manually placed 10 mm in diameter, in each hemisphere on the structural MRI for the caudate nucleus (CN), anterior putamen (AP) and posterior putamen (PP) and 8 on the occipital cortex as the reference region. Calculating each specific activity with DAT binding is estimated by the striatal-to-occipital ratio, defined as (striatum—occipital)/occipital counts. **(A)** Axial section of structural MRI (3-dimensional T1 weight image). **(B)** ROIs placed on an axial slice of MRI. **(C)** [^123^I]FP-CIT SPECT image overlaid onto the coregistered MRI.

Circular ROIs with a diameter of 10 mm were manually placed on both sides of the caudate nucleus (CN), anterior putamen (AP), and posterior putamen (PP) on transverse T1-weighted MR images, as illustrated in Figure 1 (Scherfler et al., 2005). Using the same MR image slice, the eight circular ROIs (each 10 mm in diameter) were drawn on the occipital cortex as a reference region (Figure 1). This was performed in each of the two continuous transverse slices. Higher [^123^I]FP-CIT values in the same striatal region were further selected as a specific activity. The striatal DAT binding was calculated using the striatum-occipital ratio (SOR), as shown below (Árgyelán et al., 2005).

$$\begin{array}{c} \text{SOR}=\frac{\left(\text{striatal binding}-\text{mean occipital binding}\right)}{\text{mean occipital binding}}\\ =\left(\frac{\text{striatal binding}}{\text{mean occipital binding}}\right)-1 \end{array}$$

The advantage of using this method is that six subregions of the striatum can be evaluated, although the data are not adjusted for age.

### Statistical Analysis

The normality of the variables was evaluated using Kolmogorov–Smirnov tests. Analysis 1: The relationship between SBR_adj_ and IQ was analyzed using Pearson’s correlation analysis. To clarify which WAIS-III subscores were correlated with striatal DAT binding, multiple regression analyses (stepwise linear regression analyses) were performed using SBR_adj_ values as the independent variable and the age-adjusted WAIS-III subscores as the dependent variables. Analysis 2: The relationship between age-unadjusted striatal DAT binding (SOR values) and raw WAIS-III subscores (i.e., age unadjusted) were analyzed by stepwise linear regression analyses. Analysis 3: To clarify the direct and indirect relationships between age, striatal DAT binding (SOR), and raw WAIS-III subscores, the significant results from analysis 2 were further investigated by path analyses that included age as a mediator. Path analysis is a form of multiple regression statistical analysis that is used to evaluate causal models by examining the relationships between a dependent variable. By using this method, one can estimate both the magnitude and significance of causal connections among variables. The direct path was defined as the relationship between striatal DAT binding (SOR) and WAIS-III subscore (c’), whereas the indirect path included the relationships between striatal DAT binding and age (a) and between age and WAIS-III subscores (b).

All statistical analyses were performed using IBM SPSS software version 22 (IBM Corporation, Armonk, NY, USA). A *p*-value of less than 0.05, adjusted for multiple comparisons by the false discovery rate (FDR), was considered significant. All multiple regression analyses conducted in this study showed a variance inflation factor (VIF) score of less than 1.05, excluding the possibility of collinearity between the variables.

## Results

The right and left average (± SD) striatal subregion SOR values in the 30 healthy subjects were as follows: CN: 3.50 ± 0.60; AP: 3.37 ± 0.52; and PP: 2.60 ± 0.45, thus displaying an anterior-to-posterior gradient. The average SBR_adj_ was positively correlated with the SOR of all six striatal subregions (*R* > 0.74, *P* < 0.001, Pearson’s correlation). All six striatal subdivisions (age-unadjusted SOR values) were negatively correlated with age (*R* < −0.41, *P* < 0.03).

Table 1 shows the summarized results of the WAIS-III subscores, MMSE, and FAB total scores. The following neuropsychological tests were correlated with age: coding digit symbol; block design; matrix reasoning; picture arrangement; symbol search; and FAB total score (*P* < 0.05, Pearson’s correlation adjusted by FDR; Table 1). The WAIS-III subscores for vocabulary, coding digit symbol, similarities, information, and comprehension were positively correlated with education (*R* > 0.45, *P* < 0.02).

**Table 1:** Summary of the neuropsychological tests and correlation with age and education in 30 normal subjects.

	Score [mean ± SD, (range)]	Age (*r* value^a^)	Education (*r* value^a^)
**WAIS-III**			
Full intelligence quotient (FIQ)	114.7 ± 12.9, (89–138)		
Verbal intelligence quotient (VIQ)	113.7 ± 13.6, (89–144)		
Performance intelligence quotient (PIQ)	113.1 ± 11.6, (84–132)		
Verbal comprehension	113.8 ± 12.1, (92–141)		
Working memory	106.5 ± 13.3, (74–135)		
Perceptual organization	107.1 ± 11.0, (72–128)		
Processing speed	114.8 ± 14.3, (92–151)		
**WAIS-III subscore**^b^			
Picture completion	15.9 ± 2.3, (11–19)	−0.139	0.054
Vocabulary	35.0 ± 8.7, (20–53)	0.126	0.492*
Coding digit symbol	76.8 ± 19.5, (38–111)	−0.743*	0.453*
Similarities	23.0 ± 3.8, (13–30)	−0.286	0.455*
Block design	41.3 ± 8.9, (21–57)	−0.589*	0.022
Arithmetic	13.1 ± 3.6, (7–21)	−0.275	0.324
Matrix reasoning	14.4 ± 4.7, (7–24)	−0.770*	0.412
Digit span	15.5 ± 2.9, (10– 22)	−0.184	0.051
Information	18.5 ± 3.4, (12–24)	0.014	0.563*
Picture arrangement	13.5 ± 3.9, (2–20)	−0.451*	0.211
Comprehension	20.1 ± 4.4, (13–28)	−0.171	0.488*
Symbol search	34.8 ± 8.4, (18–52)	−0.573*	0.219
Letter number sequencing	11.6 ± 2.3, (7–15)	−0.257	−0.105
Mini-mental state examination total score	29.2 ± 1.0, (26–30)	−0.188	
Frontal assessment battery total score	16.8 ± 1.6, (13–18)	−0.579*	

*P < 0.05, corrected for multiple comparisons by false discovery rate. ^a^Pearson’s correlation coefficients. ^b^Raw scores, not adjusted by age.

### Analysis 1: Age-Adjusted DAT Binding (SBR_adj_) and IQs

**Figure 2:**
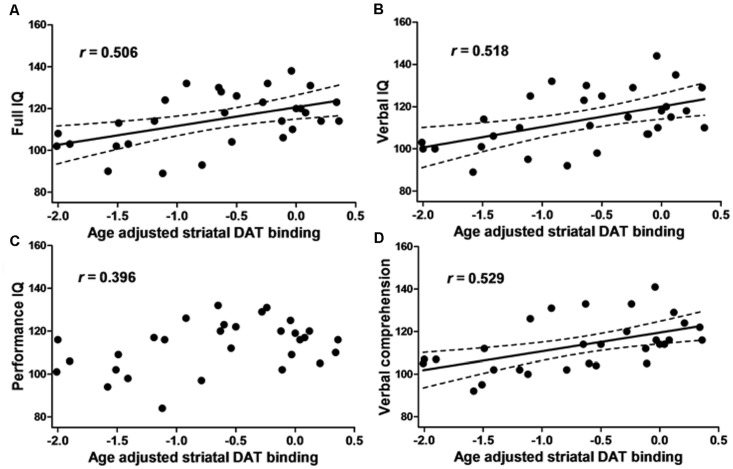
The relationship between the intelligence quotient (IQ) and dopamine transporter (DAT) binding. The scatter plot showing the age-adjusted intellectual score and age-adjusted right and left averaged striatal specific binding ratio (SBR) of [^123^I]-FP-CIT SPECT in 30 healthy subjects. **(A)** The full IQ, **(B)** Verbal IQ (VIQ) and **(D)** verbal comprehension were positively correlated with the striatal DAT binding. **(C)** Performance IQ (PIQ), working memory, perceptual organization and processing speed (see text) did not show any correlation. The dotted line represents the 95% confidence interval.

The FIQ, VIQ, and verbal comprehension scores were positively correlated with both right and left and average SBR_adj_ (right: *R* > 0.48, *P* < 0.01; left: *R* > 0.51, *P* < 0.005; average: *R* > 0.50, *P* < 0.05, Pearson’s correlations; Figure 2). PIQ, working memory, perceptual organization, and processing speed were not correlated with any SBR_adj_ values. The multiple regression analyses revealed that the right SBR_adj_ was positively correlated with the information subscore (*R* = 0.528, *P* = 0.003) and the left SBR_adj_ was positively correlated with the similarities subscore (*R* = 0.523, *P* = 0.003).

### Analysis 2: Age-Unadjusted DAT Binding (SOR Value) and Age-Unadjusted WAIS-III Subscores

The multiple regression analysis identified the WAIS-III subscores associated with each SOR; these results are shown in Table 2. The right CN SOR was positively correlated with picture completion and coding digit symbol (*R* = 0.6, *P* < 0.005). The SOR value of the left CN was positively correlated with the similarities subscore (*R* = 0.437, *P* < 0.02). The SOR value of the bilateral AP and left PP were all positively correlated with the coding digit symbol subscore (*R* > 0.43, *P* < 0.02).

**Table 2:** The multiple regression analyses of Wechsler Adult Intelligence Scale—Third Edition (WAIS-III) subscores and six subregions of striatal dopamine transporter (DAT) binding.

	Caudate		Anterior Putamen		Posterior Putamen	
	Right	Left	Right	Left	Right	Left
WAIS-III subscore	Picture completion Coding digit symbol	Similarities	Coding digit symbol	Coding digit symbol	N.S.	Coding digit symbol
*P*-value	0.002	0.016	0.011	0.004	N.S.	0.016
*R*	0.6	0.437	0.455	0.512	N.S.	0.438

*Multiple regression analyses were conducted by multiple comparisons (false discovery rate). N.S.: not significant*.

### Analysis 3: Path Analyses

**Figure 3:**
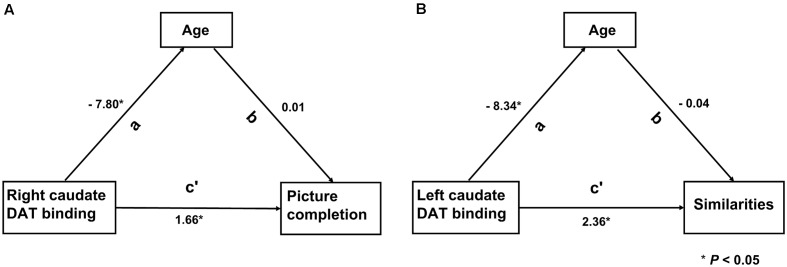
The relationship among the striatal DAT binding, Wechsler Adult Intelligence Scale—third edition (WAIS-III) subscores and age. Two models remarked significantly by path analysis. **(A)** DAT binding value of right caudate was directly associated with the WAIS-III subscores of picture completion (c’). **(B)** Left caudate was directly associated with the WAIS-III subscores of similarities (c’). The indirect path was significant (a path) in both models. In this model, the direct and indirect relationships between aging, striatal DAT binding and cognition, the standardized path coefficients (β) are listed as a, b and c’, where a and b are part of the indirect path and c’ is the direct path adjusted for the indirect path.

Six models from the results of analysis 2 were assessed, of which two models remained significant. The right CN SOR was a significant mediator of both age (*β* = −7.80, *P* = 0.013) and the picture completion subscore (*β* = 1.66, *P* = 0.013; Figure 3A). Additionally, the left CN SOR was a significant mediator of age (*β* = −8.34, *P* = 0.007) and the similarities subscore (*β* = 2.36, *P* = 0.037; Figure 3B). The caudate DAT function is both related to normal aging and cognition (picture completion and similarities), although these two relationships are independent with each other.

## Discussion

The present study investigated the physiological relationship between presynaptic striatal dopaminergic function and cognition. The following main findings were reported: (1) The FIQ, VIQ, and verbal comprehension were positively and bilaterally correlated with age-adjusted striatal DAT binding; (2) Multiple regression analyses revealed that the right age-adjusted striatal DAT binding was positively correlated with the information subscore, whereas the left age-adjusted striatal DAT binding was positively correlated with the similarities subscore; (3) Multiple regression analyses followed by path analyses revealed that age-unadjusted caudate DAT binding was a significant mediator with normal aging and with the picture completion subscore on the right and the similarities subscore on the left. This step-by-step approach demonstrated that striatal dopaminergic function, particularly involving the left CN, is implicated in language abilities in normal subjects; this relationship was independent from normal aging.

The decline in non-verbal performance with normal aging can be explained by the decreases in processing speed and problem-solving ability (fluid intelligence); conversely, verbal intelligence remained stable across age groups and is responsible for expressing previous verbal learning and general knowledge (crystallized intelligence; Ryan et al., 2000). The present study showed that coding digit symbol, block design, matrix reasoning, and picture arrangement belong to PIQ, which is an indicator of fluid intelligence and declines with age. Conversely, subscores related to verbal comprehension (i.e., similarities, information, vocabulary), known as crystallized knowledge, did not show age-related declines. These observations are in line with previous reports. Language comprehension does not decline with age; by contrast, there is an age-related decline in language production (Shafto and Tyler, 2014). This slower access to the phonological information or selective deficits in accessing phonological representations is known as “tip of tongue” state. This state may explain the dissociation between preserved knowledge and failure of semantic retrieval or bradyphrenia. A functional neuroimaging study demonstrated that retrieval failure accompanied by tip of tongue state was associated with response in the anterior cingulate and right middle frontal regions (Maril et al., 2001). Interestingly, previous findings showed that the WAIS-III information subscore in healthy subjects was not associated with aging but was correlated with striatal DAT binding, similar to our observations (Erixon-Lindroth et al., 2005). Notably, the tasks included in VIQ are not restricted by time, which indicates that the association between striatal DAT binding and VIQ cannot be explained by motor slowness.

In normal human aging, functional imaging studies of striatal DAT binding demonstrate a progressive decline of DAT expression. Previous [^123^I]FP-CIT imaging studies have reported linear normal aging effects in striatal binding with declines of 3.6%–8.1% per decade (Kazumata et al., 1998; Eusebio et al., 2012; Varrone et al., 2013; Kaasinen et al., 2015; Matsuda et al., 2018). This age-dependent decline in striatal DAT binding has been reported in humans and in primates and in rodent brain histochemical studies (Allard and Marcusson, 1989; De Keyser et al., 1990; Emborg et al., 1998; Hebert et al., 1999). A multicenter trial was conducted to generate an [^123^I]FP-CIT SPECT database of healthy controls from different SPECT cameras, in which the present subjects were part of the study (Matsuda et al., 2018). A significant effect of age was found, with a DAT binding (SBR) decline rate of 6.3% per decade. In this study, the age-unadjusted bilateral striatal SBR reported a 5.7% (CN: 7.2%, anterior 7.3%, PP: 6.2%) decline per decade, which is comparable to the values of previous reports. We have replicated the previous small-size DAT image research showing that the information subscore was related to inter-individual DAT differences (Erixon-Lindroth et al., 2005). Notably, most of the WAIS-III subscores increased in heterogeneity with aging (Ardila, 2007). This suggests that individual residuals from the age-adjusted mean may be particularly meaningful for investigating cognitive function in the process of aging. The advantage of the present study is that both cognitive function and striatal DAT binding were controlled by age, namely IQ and SBR_adj_, respectively. We propose that individual verbal ability can be explained by caudate DAT binding expressed as a deviation from the age-adjusted mean. Verbal ability is variable among subjects, and females tend to be better than males, which was first reported in a previous DAT imaging study in healthy subjects (Mozley et al., 2001). Interestingly, young women have higher striatal DAT binding than men, which is in line with our results representing the verbal aspect of striatal dopaminergic function (Matsuda et al., 2018). However, to our knowledge, most studies, including ours, are cross-sectional, and there are no longitudinal studies of DAT imaging in normal subjects. Therefore, it is difficult to reach a definitive conclusion, but we can deduce from these studies that individual striatal DAT slowly declines with normal aging, which is experienced as a decline in language function in each individual. Nonetheless, these observations lead us to the notion that language ability is associated with individual diversity of caudate dopaminergic function.

Verbal working memory is predominantly localized in the left hemisphere, whereas the spatial system localizes in the right hemisphere (Smith and Jonides, 1997). The similarity subscore, included in verbal comprehension, was related to the left-side caudate DAT function.

A meta-analysis of functional neuroimaging in healthy subjects demonstrated that the CN is coactivated with the mesial and inferior frontal gyrus, ipsilateral dorsolateral prefrontal cortex, and thalamus (Postuma and Dagher, 2006). The connectivity between the prefrontal cortex and the head of the CN is in line with the classical striato-pallido-thalamo-cortical circuits hypothesis (Alexander et al., 1990) and with evidence from a primate study (Yeterian and Pandya, 1991). Interestingly, a previous volumetric study performed in young adults showed that caudate volume was positively correlated with FIQ and VIQ but less so with PIQ; this agrees with the present results, which further supports that dopaminergic function is crucial to the association between IQ and the CN (Grazioplene et al., 2015). Likewise, the findings from this study demonstrated the relationship between cognitive function and DAT binding in the CN but not in the putamen. Neuroimaging studies that use whole-brain functional neuroimaging, semantic priming, and the neuronal adaptation technique have shown that people who can communicate in multiple languages activate the CN regardless of the type of language, which indicates that the CN is a center for language control (Crinion et al., 2006). During motor sequence acquisition in healthy subjects, perfusion images have shown activation of the bilateral caudate nuclei, the left PP, the right dentate nucleus, the ventral prefrontal cortex, the left inferior parietal lobule, and the inferior and superior temporal gyri (Carbon et al., 2003) This learning network was related to caudate DAT binding in healthy subjects (Carbon et al., 2004). The CN may play a role in prediction-error learning, which may contribute to verbal ability.

An autopsy study has found that the cellular loss of the substantia nigra subregion was prominent in the caudal subregion in normal aging (Fearnley and Lees, 1991). PD is characterized by the progressive loss of striatonigral dopaminergic neurons, which sheds light on the role of dopaminergic function in cognition. A postmortem study of patients with PD who did not show overt cognitive impairment demonstrated that the dopaminergic nerve terminals are most affected in the posterior portion of the putamen (2% of normal controls), in contrast with the relatively preserved head of the CN (19% of healthy controls; Kish et al., 1988). The ventral tier of the substantia nigra was more affected in the PD group, and this subregion projects to the dorsal putamen, which is responsible for motor function. In the same line, in non-demented PD, dopamine is predominantly depleted in the putamen with relative sparing of the CN (Kish et al., 1988). PD subjects with dementia demonstrated diminished dopaminergic function in widespread striatum, notably in the CN, compared with those without dementia (Ito et al., 2002). Accordingly, a PD-related cognitive covariance pattern demonstrated an association with dopaminergic function in the CN, which was not evident with putaminal DAT binding (Niethammer et al., 2013). Previous neuroimaging studies have repeatedly shown the pivotal role of caudate dopaminergic function and cognition in PD (Jokinen et al., 2009; Polito et al., 2012). Dopaminergic function in the CN of PD appears to be associated with verbal memory and verbal fluency and attention-demanding executive function (Marié et al., 1999; Rinne et al., 2000; Jokinen et al., 2009; Polito et al., 2012; Siepel et al., 2016). Interestingly, dopaminergic dysfunction in PD was particularly related to prefrontal cortices when investigated with glucose metabolism images (Carbon et al., 2004; Polito et al., 2012). The role of dopamine in the cognitive impairment of PD has been postulated to affect executive, memory, and visuospatial function (Niethammer et al., 2013) and language function (Mentis et al., 2002; Huang et al., 2007). Although dysfunction in the striato-frontal dopaminergic circuit is not the only pathophysiology responsible for cognitive symptoms in PD, it has a significant impact on executive and language function. These cognitive symptoms in PD subjects may potentially be modulated by dopaminergic medications.

The present study has several limitations. First, few subjects were assessed. The small number of subjects may hamper the possibility of identifying significant correlations between striatal DAT binding and cognitive function. For example, the WAIS-III information subscore was positively correlated with SBR_adj_ (*P* < 0.005) but not to achieve the subregion of striatal SOR (*P* > 0.3). In the future, increasing the sample size and diversity of cognitive tests may improve the correlation between striatal DAT binding and cognitive function. Second, not all the subjects were right-handed. However, 73% of left-handed people have left-hemisphere dominancy, so this will not strongly affect the results (Knecht et al., 2000). Third, this study had a cross-sectional design, which is insufficient to reach conclusions with regard to aging. Some of the differences noted between younger and older adults may be due to generational differences, such as differences in access to education, differences in life experiences, differences in childhood nutrition, et cetera. Fourth, gender influenced the distribution of dopamine in PD and normal subjects in a previous study (Kaasinen et al., 2015). In this study, although the male-to-female ratio was shifted (70% women), there was no gender difference in either striatal DAT binding or cognitive function (data not shown). Fifth, we assumed that the association between cognition and DAT binding was linear; however, it could be non-linear, such as an inverted U-shape, which is known to be present in the dopaminergic function, and this issue should be addressed in future studies (Cools et al., 2001). Sixth, we cannot exclude the possibility that the age-related decline of striatal dopamine was influenced by volume los by normal aging. Seventh, [^123^I]FP-CIT binds both to DAT and a little portion to serotonin transporter (Ziebell et al., 2010). Therefore, the current result may have subtle influence from the serotonin.

In conclusion, verbal function is influenced by striatal dopaminergic function, particularly by the caudate nucleus, whereas declines in non-verbal cognitive function can be explained by normal aging. This result sheds light on the association between individual deviations of striatal dopamine and language abilities in humans.

## Data Availability Statement

The raw data supporting the conclusions of this article will be made available by the authors, without undue reservation, to any qualified researcher.

## Ethics Statement

The studies involving human participants were reviewed and approved by the ethical committee of Chiba University (#2074). The patients/participants provided their written informed consent to participate in this study.

## Author Contributions

HM and SK organized the research project. SH, SF, and YN are responsible for the subjects’ recruitment and execution. SH and HL executed the research project and statistical analysis. HL wrote the first draft of the manuscript. KK, AI, and HT were responsible for the subject’s characterization and sample collection. TU, TH, and TI were responsible for scanning the subjects and supported the image technology. All authors contributed to and have approved the final manuscript.

## Conflict of Interest

The authors declare that the research was conducted in the absence of any commercial or financial relationships that could be construed as a potential conflict of interest.

## Abbreviations

DAT: dopamine transporter
FAB: frontal assessment battery
IQ: intelligence quotient
MMSE: mini-mental state examination
ROI: region of interest
SBR: specific binding ratio
SPECT: single-photon emission computed tomography
SOR: striatum-occipital ratio
WAIS-III: Wechsler Adult Intelligence Scale-Third Edition.
